# Nonuniform Cardiac Denervation Observed by ^11^C-*meta*-Hydroxyephedrine PET in 6-OHDA-Treated Monkeys

**DOI:** 10.1371/journal.pone.0035371

**Published:** 2012-04-23

**Authors:** Valerie Joers, Kailie Seneczko, Nichole C. Goecks, Timothy J. Kamp, Timothy A. Hacker, Kevin G. Brunner, Jonathan W. Engle, Todd E. Barnhart, R. Jerome Nickles, James E. Holden, Marina E. Emborg

**Affiliations:** 1 Preclinical Parkinson's Research Program, Wisconsin National Primate Research Center, University of Wisconsin-Madison, Madison, Wisconsin, United States of America; 2 Neuroscience Training Program, University of Wisconsin-Madison, Madison, Wisconsin, United States of America; 3 Department of Medicine, University of Wisconsin-Madison, Madison, Wisconsin, United States of America; 4 Department of Medical Physics, University of Wisconsin-Madison, Madison, Wisconsin, United States of America; Technical University of Dresden Medical School, Germany

## Abstract

Parkinson's disease presents nonmotor complications such as autonomic dysfunction that do not respond to traditional anti-parkinsonian therapies. The lack of established preclinical monkey models of Parkinson's disease with cardiac dysfunction hampers development and testing of new treatments to alleviate or prevent this feature. This study aimed to assess the feasibility of developing a model of cardiac dysautonomia in nonhuman primates and preclinical evaluations tools. Five rhesus monkeys received intravenous injections of 6-hydroxydopamine (total dose: 50 mg/kg). The animals were evaluated before and after with a battery of tests, including positron emission tomography with the norepinephrine analog ^11^C-*meta*-hydroxyephedrine. Imaging 1 week after neurotoxin treatment revealed nearly complete loss of specific radioligand uptake. Partial progressive recovery of cardiac uptake found between 1 and 10 weeks remained stable between 10 and 14 weeks. In all five animals, examination of the pattern of uptake (using Logan plot analysis to create distribution volume maps) revealed a persistent region-specific significant loss in the inferior wall of the left ventricle at 10 (*P*<0.001) and 14 weeks (*P*<0.01) relative to the anterior wall. Blood levels of dopamine, norepinephrine (*P*<0.05), epinephrine, and 3,4-dihydroxyphenylacetic acid (*P*<0.01) were notably decreased after 6-hydroxydopamine at all time points. These results demonstrate that systemic injection of 6-hydroxydopamine in nonhuman primates creates a nonuniform but reproducible pattern of cardiac denervation as well as a persistent loss of circulating catecholamines, supporting the use of this method to further develop a monkey model of cardiac dysautonomia.

## Introduction

Parkinson's disease (PD) is a movement disorder associated with degeneration of the nigrostriatal dopaminergic pathway; however, patients also experience nonmotor symptoms, with up to 80% having some form of autonomic dysfunction [1], [2], [3], [4]. Dysautonomias greatly affect PD patients' quality of life, and often are more disabling than motor deficits [5].

Cardiac dysautonomia, or abnormal autonomic control of the heart, is characterized in PD by orthostatic hypotension, an increase in corrected QT intervals (QTc), and reductions in heart rate variability and plasma norepinephrine [6], [7], [8], [9], [10]. Cardiac denervation, which may be a component of dysautonomias or found independently in PD patients, [11], [12], [13], [14] is associated with arrhythmias, shortness of breath during exercise, reduced time to peak heart rate, and fatigue [6], [15]. High co-prevalence suggests cardiac denervation and dysautonomia are intimately related with serious clinical consequences. New screening tools [7] and treatments are needed.

Cardiac sympathetic loss in PD has been documented using sympathoneuronal imaging agents such as 6-[^18^F]fluorodopamine [11], [16], [17], [18] and ^11^C-*meta*-hydroxyephedrine (MHED) [19], [20] with positron emission tomography (PET) and [^123^I]metaiodobenzylguanidine with single-photon emission computed tomography [12], [13], [21], [22], [23]. Low uptake in the left ventricle (LV) myocardium is reported in all stages of PD, although specific patterns of loss are not well described. Both diffuse and localized losses to lateral and inferior LV walls are reported [12], [20], [24]. Regardless of the pattern, loss is progressive [6]. PD patients exhibited as much as 30% loss of 6-[^18^F]fluorodopamine uptake in the LV lateral wall over 2 years, with relative preservation in the septal wall [18]. Loss of cardiac sympathetic innervation is confirmed post mortem by nearly complete absence of tyrosine hydroxylase, an enzyme in catecholamine biosynthesis, and by reduced neurofilament protein, a marker for the presence of axons [25], [26], [27].

Development of PD treatments targeting cardiac dysautonomia requires comprehensive animal models mimicking this symptom, [28] but no well-characterized nonhuman primate model of cardiac dysautonomia is currently available. Systemic administration of 6-hydroxydopamine (6-OHDA) to mice [29], [30] and dogs [31], [32] induces persistent loss of cardiac catecholaminergic innervation, supporting use of 6-OHDA to model cardiac dysautonomias in PD. We therefore aimed to assess the feasibility of developing a monkey model of cardiac dysautonomia by administering 6-OHDA within a system of *in vivo* evaluation in order to generate preclinical tools toward understanding this condition and testing therapies for PD patients.

## Materials and Methods

### Ethics Statement

The present study was performed in strict accordance with the recommendations in the NIH Guide for the Care and Use of Laboratory Animals (7^th^ edition, 1996) in an AAALAC accredited facility (Wisconsin National Primate Research Center, University of Wisconsin - Madison). Experimental procedures were approved by the Institutional Animal Care and Use Committee of the University at the Wisconsin-Madison (permit number: G00538). All efforts were made to minimize the number of animals used and to ameliorate any distress.

### Subjects

**Figure 1:**
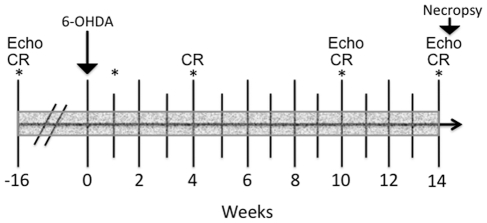
Experimental timeline. All procedures were performed in 5 adult rhesus monkeys following a within-subject experimental design. MHED PET scans, plasma catecholamines, troponin I and ECGs (*) were performed at baseline and 1, 4, 10 and 14 weeks after 6-OHDA. Echocardiograms (Echo) were obtained at baseline and 10 and 14 weeks after 6-OHDA. Clinical rating (CR) was performed at baseline and 4, 10 and 14 weeks following toxin administration. Food consumption and feces were monitored daily and body weight was measured weekly throughout the course of the study (shading). Animals were euthanized 14 weeks following 6-OHDA administration.

Five adult rhesus monkeys (*Macaca mulatta*; 5–9 years old; 6–10 kg; two male, three female) were used in this project. The study followed a within-subjects experimental design (Figure 1) in which each animal was used as its own control. This design facilitates matching subjects between groups, reduces the chance of confounding factors, and the number of monkeys used. The animals were individually housed in Group 3 or Group 4 enclosures (cage floor area 4.3 ft.2 or 6.0 ft.2 per animal, height 30 or 32 in.) in accordance with the Animal Welfare Act and its regulations and the 7th edition of the Guide for the Care and Use of Laboratory Animals (1996) with a 12-hour light/dark cycle. Throughout the study, the animals were monitored twice daily by an animal research technician or veterinary technician for evidence of disease or injury (e.g., inappetance, dehydration, diarrhea, depression, lethargy, trauma, etc.) and body weight was documented weekly to ensure animals remained in properly sized cages. Animals were fed commercial nonhuman primate chow (2050 Teklad Global 20% Protein Primate Diet, Harlan Laboratories, Madison, WI) twice daily, supplemented with fruits or vegetables and a variety of forage items and received *ad libitum* water. Nonhuman primate chow soaked in a protein-enriched drink (Ensure©, Abbott Laboratories, Abbott Park, IL) was offered to stimulate appetite as needed after neurotoxin dosing.

### 6-OHDA Dosing

6-OHDA hydrobromide (Sigma-Aldrich, St. Louis, MO) solution was prepared under a certified chemical hood, less than 2 hours before administration. The solution was kept out of light and on ice until immediately before administration, when it was drawn up into a 10-mL syringe covered with foil, and flushed through an amber-colored infusion line. Animals were food-deprived overnight; anesthesia was induced with ketamine HCl (15 mg/kg im) and maintained with 1–3% isoflurane in 100% O_2_ at 1 L/min. 6-OHDA (total final dose 50 mg/kg) was mixed into a sterile ascorbic acid solution (1 mg/mL 0.9% NaCl) and administered intravenously in a series of 5 mL injections (see Table 1 for dosing scheme) at a rate of 1 mL/min, using a motorized syringe pump (KD Scientific, Holliston, MA). Dosing was based on previous reports administering 6-OHDA to dogs [31], [32]. Blood pressure, heart rate, respiration rate, blood oxygen, and electrocardiograms (ECG) were monitored throughout the procedure; their normalization (return to baseline values) defined timing of subsequent dosing. Before dosing, blood was taken for complete blood chemistry and hematocrit measurements; additional hematocrit measurements were performed after each dosing.

**Table 1:** 6-OHDA dosing scheme (mg/kg) for each individual animal.

Injection number	R01097	R01098	R04094	RH2316	RH2318
1	0.50	0.50	0.50	0.49	0.50
2	1.00	1.00	1.00	1.00	1.00
3	1.50	0.62	1.50	2.30	1.50
4	2.00	0.88	2.00	0.85	2.00
5	4.00	2.00	4.00	5.00	4.00
6	15.00	4.00	15.00	10.00	10.00
7	26.00	10.00	26.00	11.84	10.00
8		10.00		18.52	21.00
9		21.00			
Total	50.00	50.00	50.00	50.00	50.00

The rhesus monkeys received 7–9 injections of 6-OHDA solution in one session, accumulating to a total final dose of 50 mg/kg. Dosing and timeline of injections varied based on the individual reaction to 6-OHDA. The normalization (return to baseline measures) of vital signs, ECG, heart rate, and blood pressure was used to define when the next dose could be given.

### Clinical Evaluations

Heart rate, cardiac auscultation, complete blood count, and blood chemistry were performed at least monthly before and after 6-OHDA. Feces were monitored daily by trained personnel and their characteristics recorded using a descriptive scale. The feces were identified as: no stool, very little stool, firm stool, soft feces (feces not-formed, usually soft or loose), diarrhea (watery or fluid feces), and mucus feces.

A trained observer monitored for PD signs using a previously validated clinical rating scale [33], [34]. The scale rates tremor (0–3 for each arm), posture (0–2), gait (0–5), bradykinesia (0–5), balance (0–2), gross motor skills (0–4 for each arm), defense reaction (0–2) and freezing (0–2). The total score ranges from 0 (normal condition) to 32 points (extreme severe disability).

A 10-lead ECG (Hewlett-Packard PageWriter, MA) was performed at baseline, during toxin administration, and at 1, 4, 10, and 14 weeks after 6-OHDA. Data were collected at least 15 minutes after starting isoflurane anesthesia. Electrodes were placed on identical positions on the interior right and left arms and legs, the 4th intercostal space immediately right of the sternum (V3), the 5th intercostal space immediately left of the sternum, and directly across from V3 at the anterior axillary line.

An echocardiogram (LOGIQe; GE Healthcare, Waukesha, WI) was performed under 1–3% isoflurane anesthesia in 100% O_2_ (1 L/min) at baseline and at 10 and 14 weeks after 6-OHDA. Measurements included heart rate, wall thickness, LV chamber diameter, fractional shortening (FS), velocity of blood through mitral and aortic valves, isovolumetric relaxation time, and aortic diameter.

### Troponin I and Catecholamines Analysis

Blood samples for plasma troponin I and catecholamines were obtained at baseline and at 1, 4, 10, and 14 weeks after toxin administration. Blood was collected in a K_2_ EDTA tube immediately mixed with 10% sodium metabisulfite (0.7%) and centrifuged. For plasma troponin I, samples were analyzed by an enhanced sensitivity immunometric immunoassay as per manufacture instructions (VITROS Immunodiagnostic Products). Plasma norepinephrine, dopamine and epinephrine and their deaminated metabolite dihydroxyphenylacetic acid (DOPAC) were assayed by high-performance liquid chromatography (HPLC) with electrochemical detection (ESA, Chelmsford, MA). Two animals (RH2318 and RH2316) had baseline catecholamines drawn under ketamine and medetomidine, an adrenergic agonist, and were removed from individual catecholamine analysis. A1.0 mL of plasma was analyzed using a coulometric electrochemical detector (Choulochem III; ESA, Chelmsford, MA). Every 1 L of mobile phase used to separate the catecholamines contained 13.8 g of sodium phosphate, 55 mg of 1-octane sulfonic acid, 55 mg of EDTA and 45 mL of acetonitrile at pH of 3.85 and filtered through a 0.22 µm GV filter under vacuum and pumped into the system at a rate of 0.6 mL/min producing a pressure approximately 69–71 bars. The electrodes were set at −250 and 380 mV. Peak heights were measured from the chromatograms and original concentrations of the plasma sample were determined by correcting for the incomplete recovery relative to the internal standards. Coefficients of variation between standards were at acceptable levels for norepinephrine (0.014), epinephrine (0.023), dopamine (0.015) and DOPAC (0.014). For statistical analysis, levels below detectable HPLC sensitivity, were considered as the threshold value (e.g.: epinephrine, 3.0 mg/mL; norepinephrine, 1.5 pg/mL; DOPAC and epinephrine assays were all within detectable ranges).

### Radiosynthesis of C^11^ *Meta*-hydroxyephedrine (MHED)

MHED was prepared by UW-Madison Medical Physics cyclotron facility using published methods [35]. Briefly, [11C]MeI was produced from in-target [11C]CH4 on a 16 MeV GE PETtrace using a Scansys automated chemistry module. Gas phase [11C]MeI labeled 15 mg metaraminol free base (ABX, Germany) in 300 µL 1∶2 DMSO∶DMF. The sealed vial was heated to 110°C for 5 minutes and diluted with 5 mL water for preparative reverse-phase HPLC purification (Econosil 5 µ, 8×300 mm, 3 mL/min 0.1 M Na2HPO4, with EtOH concentration increasing linearly from 0 to 10% over 600 s). The final product was millipore filtered for injection.

### [^11^C]MHED PET Imaging

Monkeys underwent MHED PET under isoflurane anesthesia (1–3% in 100% O_2_, 1 L/min) at baseline and at 1, 4, 10, and 14 weeks after 6-OHDA. Animals were positioned supine in a Siemens HR+ PET scanner, using a custom-made foam positioning apparatus. After a 15 minute transmission scan, MHED was injected as an intravenous bolus (≤5.2 mCi) over 30 s. Dynamic PET images were obtained for 1 hour with conventionally increasing frame durations (6×30s, 3×60s, 2×120s, 10×300s).

### PET Data Analysis

Whole-blood tracer concentrations were obtained from a volume of interest in the upper part of the LV chamber. MHED uptake was quantified using the equilibrium distribution volume (DV) [36] of the LV tissue relative to whole blood. A DV value of 1 corresponds to no excess capacity in tissue relative to blood; DV – 1 thus provides a measure of the density of the nerve terminals that give the tissue its excess capacity. DV was evaluated in eight sectors in each of seven short-axis rings evenly spaced along the long axis of the heart. Data are presented as polar maps (apex of the heart at the center, base of the LV at the edge). Intersubject variation was observed at baseline in the apical and basal regions, probably reflecting normal patterns of innervation. Study of one animal (R01097) at the 10-week time point failed for technical reasons; uptake patterns at other time points were similar to those of the other four animals and were included in the visual assessments. Statistical assessments of PET data were performed with only the four remaining animals.

Individual subject global DV was calculated as an average over all eight sectors from polar rings 2 to 6 (total: 40 blocks) at baseline and at 1, 4, 10, and 14 weeks after lesion. These measures were averaged across animals to determine the group global DV at each timepoint. Individual MHED percent retention deficit was calculated for each of the 40 blocks at each time point as [(DV_baseline_−1)−(DV_timepoint_−1)]/(DV_baseline_−1)×100 and averaged over blocks at each time point. These measures also were averaged across animals to obtain group MHED percent retention deficit at the four post-lesion time points.

### Statistical Analysis

All statistical analysis was performed using GraphPad Prism (version 5.0b, GraphPad Software). A *P*<0.05 was accepted as significant. Comparison over time of the different data sets (feces, weights, ECG intervals, echocardiogram data, troponin I and catecholamines levels) were done using repeated measures ANOVA and corrected with Bonferroni multiple comparison tests.

Changes over time of global DV mean and percent retention deficit were assessed by repeated-measures ANOVA and corrected with Tukey's multiple comparisons test. To assess regional variation, individual DV values in the four single sectors in the anterior, septal, inferior, and lateral directions were averaged over the five central rings (total: 5 blocks) to provide a single mean regional value at each time point. Group averaged regional DV values were analyzed with two-way repeated-measures ANOVA among all four regions over time and corrected with Bonferroni multiple comparisons test. Finally, Pearson correlations were performed between MHED uptake and other outcome measures (feces, ECG, blood assays and echocardiogram outputs) and between body weights and frequency of abnormal feces.

## Results

### Immediate Effects of 6-OHDA Administration

**Figure 2:**
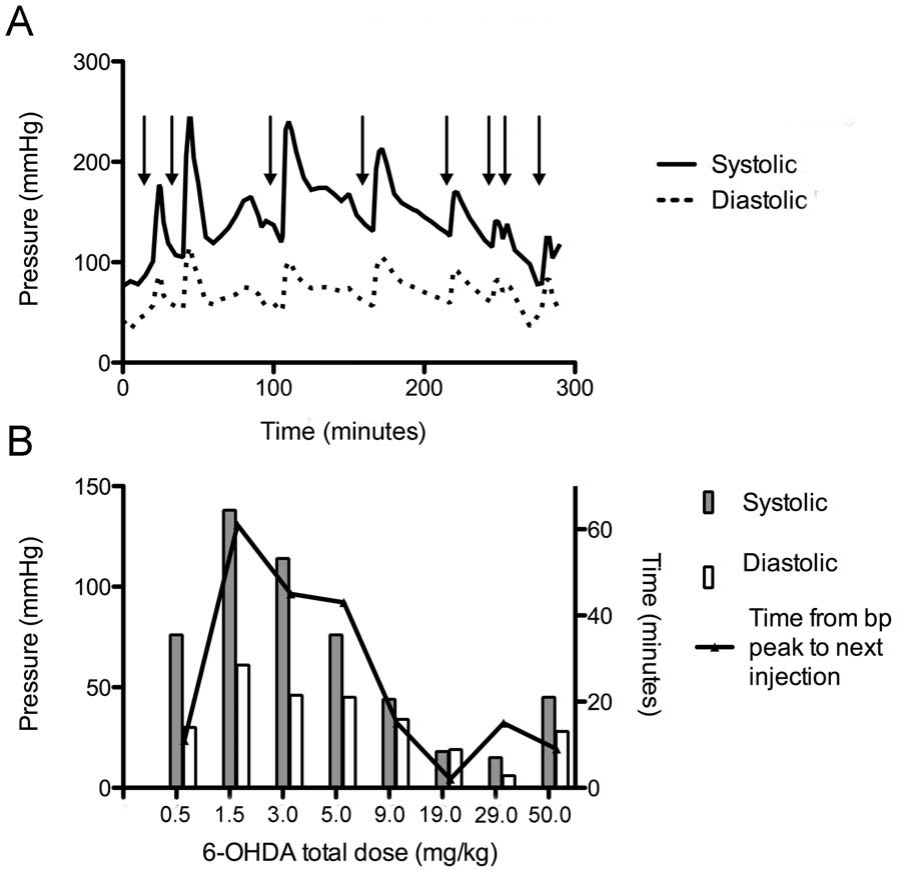
Systolic and diastolic blood pressure measurements during 6-OHDA administration. Blood pressure results from a representative animal (RH2318). A, Both pressures increase immediately after each 6-OHDA dosing (arrows). B, Change in systolic and diastolic blood pressure from peak of pressure following 6-OHDA until levels normalized at which time the next injection of 6-OHDA was administered. The amount of time to normalize paralleled the change in blood pressure. At the accumulated dose of 2.0 mg/kg, this animal required 61 minutes to regulate blood pressure. bp, blood pressure.

Sympathetic responses occurred immediately after each 6-OHDA injection, including increases in systolic and diastolic blood pressure (Figure 2*A*) and heart rate. Individual animal sensitivity and the tachyphylaxic effect of sympathetic responses to 6-OHDA dosing influenced the time interval between successive doses that ranged from 12 to 100 minutes with the longest period occurring after a cumulative dose of >1.5–3 mg/kg (Table 1; Figure 2*B*).

In three animals (R01098, R04094, RH2316), ECGs showed arrhythmic activity (e.g., ventricular premature contractions) when the cumulative dose reached >3 mg/kg, which delayed next dosing. Increasing the number of injections (while decreasing the amount of 6-OHDA per injection) and adjusting each dose according to the individual response minimized side effects and reduced time between consecutive doses.

Hematocrit levels were slightly elevated during toxin delivery; peak increases averaged 18.1%. Three animals (R01097, R01098, R04094) vomited during or directly after treatment. All animals produced pink-hued urine after 6-OHDA, probably because of oxidation of 6-OHDA; samples were negative for blood.

### Clinical Effects of 6-OHDA Administration

Group weekly weights showed body weight loss after 6-OHDA compared to baseline. The weight loss became statistically significant 4 weeks after lesion (12.26%) and remained stable over time (Table 2).

**Table 2:** Weekly animal weights (kg) and average group % weight loss before and after 6-OHDA dosing.

Animal ID	Baseline	1	2	3	4	8	10	11	12	13	14
R01097	8.38	7.98	7.96	8.14	7.55	7.68	7.36	7.51	7.09	7.29	7.24
R01098	6.44	5.86	5.89	6.00	5.63	5.48	5.52	5.71	5.47	5.66	5.57
R04094	8.00	7.22	7.28	7.32	6.74	6.42	6.28	6.58	6.21	5.99	5.94
RH2316	9.60	9.70	8.35	8.33	8.33	8.38	8.50	8.43	8.70	8.75	8.67
RH2318	9.55	8.49	8.53	8.55	8.61	8.99	9.12	9.32	9.05	9.50	9.34
Average (kg)	8.39	7.85	7.60	7.67	7.37**	7.39**	7.36**	7.50*	7.30**	7.44*	7.35**
SEM	0.58	0.64	0.48	0.47	0.54	0.64	0.67	0.64	0.69	0.75	0.74
Average group % loss		6.72	9.25	8.40	12.26	12.32	12.78	10.84	13.49	11.92	12.95

A significant loss of weight was found starting at four weeks post 6-OHDA challenge and persisting throughout the study.

* *P*<0.05,

** *P*<0.01.

Soft feces or diarrhea were observed after 6-OHDA dosing but did not interfere with general and motor behavior as well as blood hematology and chemistry. The frequency of abnormal feces positively correlated with body weight loss (Pearson's correlation, r^2^ = 0.516, *P*<0.0001), in particular diarrhea (r^2^ = 0.3604, *P* = 0.0005) and soft feces (r^2^ = 0.4015, *P* = 0.0002).

ECGs after 6-OHDA all demonstrated normal sinus rhythm. There were no significant changes in PR, QRS and QTc intervals relative to baseline, despite QTc being considered an indicator of cardiac dysautonomia in Parkinson's disease (Table 3) [37], [38].

**Table 3:** Electrocardiogram (ECG) measurements at baseline, 1, 4, 10 and 14 weeks after 6-OHDA for each animal.

Animal ID	Time	HR (bpm)	PR (ms)	QRS (ms)	QT (ms)	QRS axis	ΔQTc
RH2316	Baseline	140	80	45	230	84	
	1 wk	112	90	77	296	65	53.10
	4 wk	112	90	40	240	110	−23.40
	10 wk	130	105	50	250	110	16.70
	14 wk	135	90	48	242	110	11.70
RH2318	Baseline	130	78	50	260	45	
	1 wk	125	80	48	265	30	−0.21
	4 wk	142	95	40	240	45	−13.51
	10 wk	148	82	38	280	45	57.09
	14 wk	135	100	44	285	45	44.79
R01097	Baseline	132	90	50	240	50	
	1 wk	129	90	45	280	45	54.60
	4 wk	165	85	43	240	45	42.00
	10 wk	140	90	47	235	45	33.40
	14 wk	137	100	46	245	45	6.70
R01098	Baseline	120	92	45	295	45	
	1 wk	135	95	54	270	80	−12.20
	4 wk	145	90	55	290	45	33.60
	10 wk	138	98	54	269	45	20.20
	14 wk	134	89	51	300	45	21.70
R04094	Baseline	140	98	48	250	65	
	1 wk	120	104	50	315	65	63.60
	4 wk	140	100	51	260	85	15.30
	10 wk	132	90	54	280	90	−9.20
	14 wk	145	90	50	250	85	31.10

QTc was calculated using the Bazett's formula as QT interval/sqrt (RR interval), where the RR interval was calculated 60/HR. ΔQTc was calculated as QTc of each time point minus baseline. No significant differences were found in PR, QRS and QTc intervals obtained in anesthetized animals. HR, heart rate. QTc, corrected QT interval.

Echocardiograms revealed variable changes in FS, a measure of systolic performance following 6-OHDA (Table 4). Three animals had a decrease in FS throughout the study with the largest decrease being 43% from baseline. In two of these three animals, there was left ventricular dilation accompanying the systolic dysfunction. Two animals showed no apparent changes in chamber size or FS. There were no notable clinical consequences observed from FS reductions such as signs of heart failure. Evaluation of wall thickness, did not reveal significant changes associated to nonuniform wall innervation (Table 4).

**Table 4:** Echocardiogram measurements at baseline, 10 and 14 weeks after 6-OHDA for each animal.

Animal ID	Time	AWd (cm)	AWs (cm)	PWd (cm)	PWs (cm)	LVD_d_ (cm)	LVD_s_ (cm)	FS	FS (% change from baseline)
RH2316	Baseline	0.48	0.86	0.44	1.01	2.10	0.94	55%	
	10 wk	0.44	0.85	0.44	0.95	2.20	1.08	51%	−8%
	14 wk	0.45	0.96	0.45	0.96	2.22	0.96	57%	+3%
RH2318	Baseline	0.47	0.88	0.50	1.08	2.35	1.28	46%	
	10 wk	0.48	0.76	0.52	0.82	2.37	1.75	26%	−43%
	14 wk	0.48	0.86	0.49	0.95	2.52	1.60	37%	−20%
R01097	Baseline	0.47	0.81	0.48	0.75	2.01	0.94	53%	
	10 wk	0.49	0.82	0.47	0.74	2.17	1.10	49%	−7%
	14 wk	0.51	0.89	0.48	0.81	2.19	1.35	38%	−28%
R01098	Baseline	0.46	0.93	0.48	0.78	2.06	1.16	44%	
	10 wk	0.45	0.77	0.45	0.72	1.97	1.16	41%	−6%
	14 wk	0.44	0.82	0.40	0.72	1.98	1.11	44%	+1%
R04094	Baseline	0.46	0.77	0.49	0.82	2.16	1.19	45%	
	10 wk	0.45	0.86	0.42	0.86	2.15	1.12	48%	+7%
	14 wk	0.48	0.87	0.42	0.71	2.02	1.32	35%	−23%

FS is calculated as [(LVD_d_ – LVD_s_)/LVD_d_]×100. Three animals experienced large decreases in FS compared to baseline, but there was no significant change in left ventricle diameter or in anterior and posterior wall thickness. This suggests that the increased luminal dimensions of the left ventricle were not due to the loss of cardiac muscle. AWd, anterior wall thickness in diastole. AWs, anterior wall thickness in systole. PWd, posterior wall thickness in diastole. PWs, posterior wall thickness in systole. LVD_d_, left ventricle diameter in diastole. LVD_s_, left ventricle diameter in systole. FS, fractional shortening.

### Blood Assays

Troponin I levels in four animals were not affected by 6-OHDA. One animal (R04094) had a fivefold increase at 1 week, suggestive of cardiac damage; levels returned to baseline over time.

Plasma catecholamine levels dramatically decreased 1 week after 6-OHDA and remained stable until necropsy (Table 5), with significant reduction in norepinephrine (*F*_2,4_ = 6.57, *P* = 0.012) and DOPAC (*F*_2,4_ = 17.20, *P* = 0.0005) levels. Decreases in dopamine and epinephrine did not reach statistical significance, probably because of individual variability at baseline.

**Table 5:** Circulating plasma catecholamine levels (pg/mL) at baseline (0) and at 1, 4, 10, and 14 weeks after 6-OHDA in three animals.

Animal ID	Time	Norepinephrine pg/mL (ln)*	Epinephrine pg/mL (ln)	Dopamine pg/mL (ln)	DOPAC pg/mL (ln)**
R01097	0	22.35 (3.11)	10.33 (2.34)	13.25 (2.58)	79.64 (4.38)
	1	3.61 (1.28)	3.83 (1.34)	1.74 (0.56)	26.48 (3.28)
	4	4.73 (1.55)	4.08 (1.41)	1.26 (0.23)	13.52 (2.60)
	10	3.85 (1.35)	nd	1.31 (0.27)	14.46 (2.67)
	14	nd	nd	2.77 (1.02)	21.29 (3.06)
R01098	0	89.92 (4.50)	102.19 (4.63)	86.98 (4.47)	103.77 (4.64)
	1	4.18 (1.43)	3.20 (1.16)	2.02 (0.70)	14.99 (2.71)
	4	4.50 (1.50)	6.02 (1.80)	1.36 (0.31)	12.50 (2.53)
	10	nd	4.11 (1.41)	3.55 (1.27)	12.13 (2.50)
	14	4.81 (1.57)	5.24 (1.66)	2.82 (1.04)	17.15 (2.84)
R04094	0	49.06 (3.89)	20.96 (3.04)	18.49 (2.92)	56.92 (4.04)
	1	4.25 (1.45)	1.87 (0.63)	3.57 (1.27)	16.82 (2.82)
	4	3.91 (1.36)	3.35 (1.21)	1.03 (0.03)	16.21 (2.79)
	10	2.26 (0.81)	3.43 (0.81)	2.49 (0.91)	19.04 (2.95)
	14	4.39 (1.48)	nd	7.12 (1.96)	21.47 (3.07)

The values in parentheses are the natural logarithms of the concentration values and used to graph correlations in Figure 5. For statistical analysis, non-detectable levels (nd) levels were considered as the lowest detection level with 1.5 pg/mL for norepinephrine and 3.0 pg/mL for epinephrine. Post hoc analysis with Bonferroni multiple comparisons detected significance at all timepoints compared to baseline for norepinephrine and DOPAC (*P<0.05, ** *P*<0.01).

### Analysis of MHED Uptake

**Figure 3:**
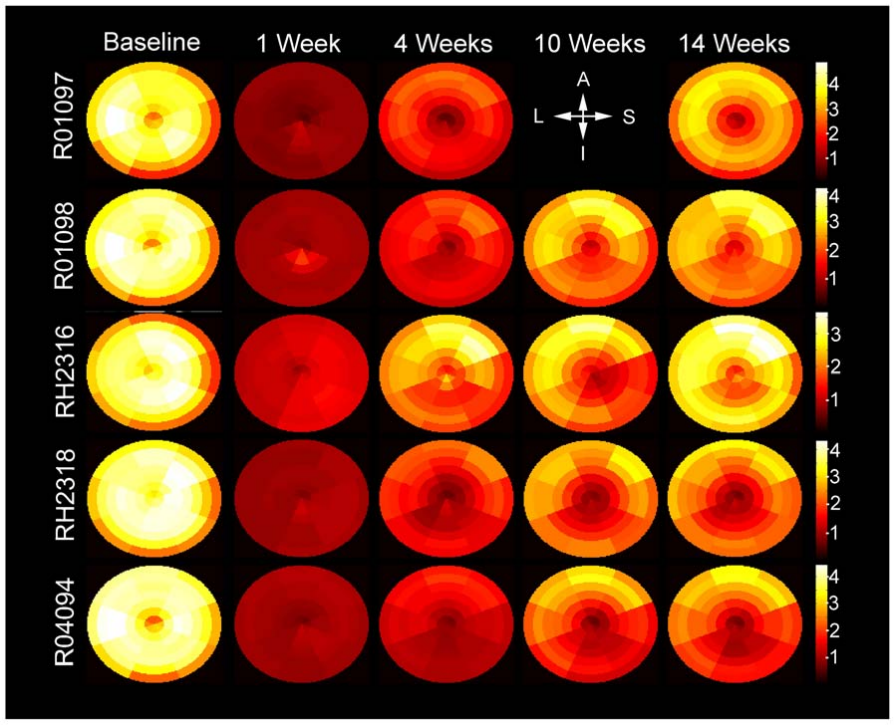
Distribution volume maps before and after systemic 6-OHDA dosing. Maps express regional capacity of the tissue for MHED uptake relative to whole blood, thus providing a measure of the density of nerve terminals. Each individual map consists of 8 sectors and 7 rings (apex of the heart at the center, base of the LV at the edge), totaling to 56 blocks of data per timepoint. MHED uptake significantly decreased at each timepoint after 6-OHDA compared to baseline (*P*<0.001). Scales (mL whole blood/g tissue) are similar between animals and identical across time points for each individual. The compass rose indicates regions: A, anterior; S, septal; I, inferior; L, lateral.

**Figure 4:**
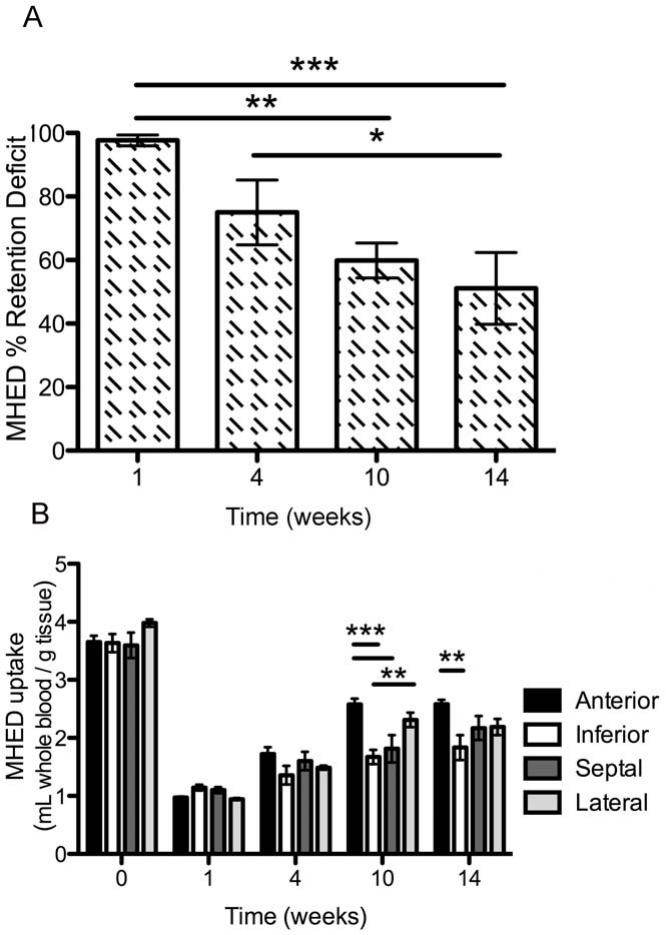
Quantitative analysis of MHED PET. A, Group mean global MHED % retention deficit comparing 1, 4, 10, and 14 weeks after toxin to baseline. B, Group mean regional MHED uptake comparing anterior, inferior, septal, and lateral regions of the left ventricle at baseline and at 1, 4, 10, and 14 weeks after toxin. *P<0.05, ** *P*<0.01, *** *P*<0.001.

Baseline DV values were uniform, regionally and across animals. DV values fell to ∼1 in all regions in all animals at 1 week after 6-OHDA, and slowly recovered over 3 months with considerable regional variation (Figure 3). Group-averaged global DV values at each time point after 6-OHDA were all significantly lower than at baseline (*P*<0.001), suggesting reduction of innervation 3 months after 6-OHDA. Similarly, group MHED percent retention deficit values varied significantly over time (*F*_3,3_ = 14.38, *P*<0.001), with reductions noted between 1 and 10 weeks (*P*<0.01), 1 and 14 weeks (*P*<0.001), and 4 and 14 weeks after toxin (*P*<0.05) but not between directly adjacent time points (Figure 4*A*). The continual increase in MHED uptake suggests slow but global recovery of innervation; however, significant changes were detected between 10 and 14 weeks after toxin challenge, suggesting stabilization of the lesion.

Statistical analysis demonstrated a main effect on each variable region (*F*_3,48_ = 5.93, *P*<0.05) and time (*F*_4,48_ = 231.4, *P*<0.001), in addition to the interaction effect (*F*_12,48_ = 2.986, *P*<0.01). *Post hoc* analysis between regions over time showed a significant difference between anterior and inferior regions at 10 (*P*<0.001) and 14 weeks (*P*<0.01), anterior and septal at 10 weeks (*P*<0.001), and inferior and lateral at 10 weeks (*P*<0.01), suggesting that 6-OHDA creates a heterogeneous lesion in the LV myocardium (Figure 4*B*).

**Figure 5:**
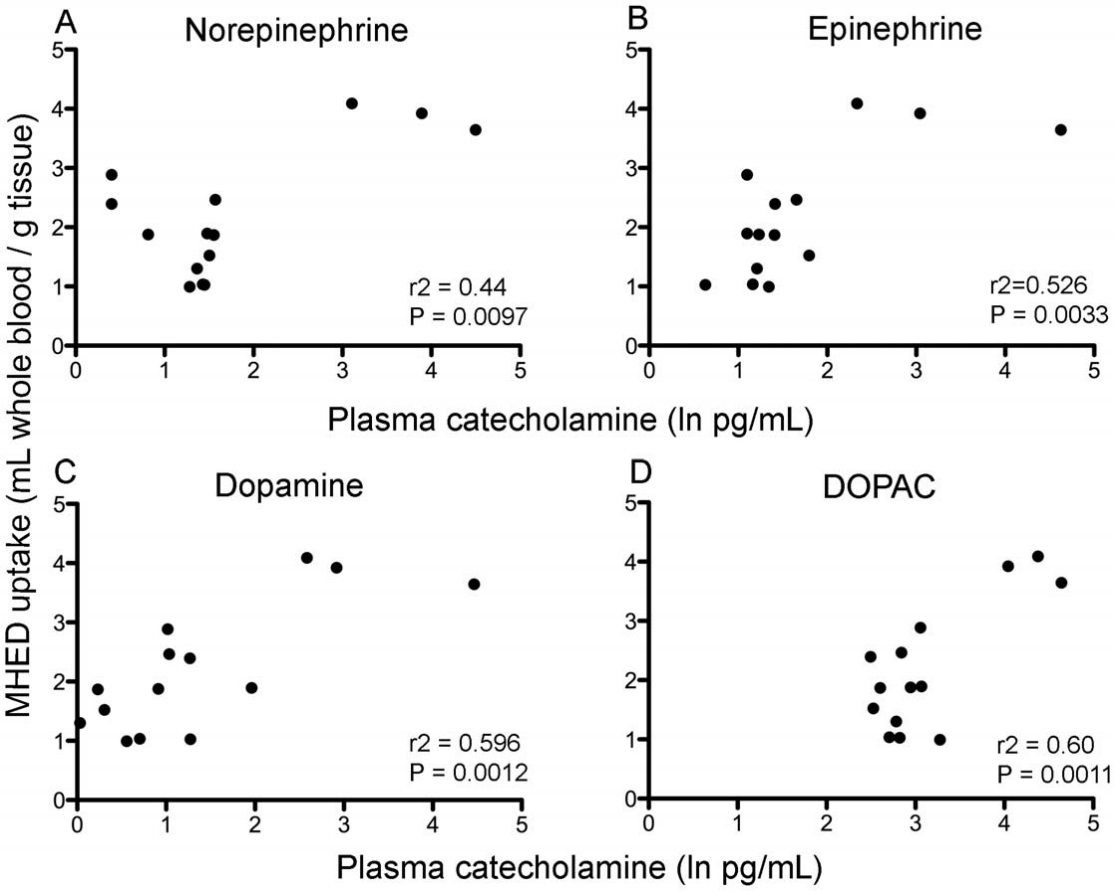
Correlation between plasma catecholamine levels and MHED uptake. Significant correlations between normalized blood catecholamine concentrations and left ventricle myocardium MHED uptake. A, Norepinephrine. B, Epinephrine. C, Dopamine. D, DOPAC.

Cardiac MHED uptake was significantly correlated with the natural logarithm of the blood concentrations of norepinephrine (Pearson's correlation; *r^2^* = 0.44, *P* = 0.0097), dopamine (*r^2^* = 0.596, *P* = 0.0012), epinephrine (*r^2^* = 0.526, *P* = 0.0033), and DOPAC (*r^2^* = 0.60, *P* = 0.0011) at similar time points (Figure 5). Additionally, frequency of abnormal feces significantly corresponded with MHED uptake (r^2^ = 0.4814, *P* = 0.0059). No significant correlations were found between MHED and troponin I or FS changes.

## Discussion

To our knowledge, this is the first study to characterize *in vivo* effects in the heart of systemic 6-OHDA to rhesus monkeys, and the first to track experimental cardiac denervation with MHED PET. The main findings include a nonuniform pattern of MHED uptake in the left myocardium, as well as decrease in circulating catecholamines, suggesting that this model mimics cardiac dysautonomia in PD.

The most common nonhuman primate model of PD involves the neurotoxin 1-methyl-4-phenyl-1,2,3,6-tetrahydropyridine (MPTP) [39]. Systemic dosing of MPTP produces a severe parkinsonian motor syndrome; however, effects in sympathetic denervation are temporary, as observed with 6-[^18^F]fluorodopamine PET imaging [40]. The other frequently used PD monkey model depends on direct intracerebral delivery of 6-OHDA, in order to bypass the blood–brain barrier and affect the nigrostriatal pathway without inducing peripheral effects [39]. Our method of intravenous delivery takes advantage of 6-OHDA catecholaminergic toxicity to develop a cardiac dysautonomia model.

The only previous report of systemic administration of 6-OHDA to monkeys was done in a single rhesus to compare the systemic effect of 6-OHDA to MPTP [40]. Yet, the authors did not describe the 6-OHDA method of administration and only provided a 6-[^18^F]fluorodopamine PET scan image and catecholamine levels 1 week after intoxication. Instead, we based our dosing regime on the reports of 6-OHDA administration to dogs, which described neurotoxin delivery over several hours to avoid clinical complications associated to the dopaminomimetic effects of the neurotoxin [31], [32]. Intravenous administration of 6-OHDA proved to require intensive monitoring and veterinary care to avoid hypertensive crisis and pulmonary edema. The adjustment of the dosing regimen to the individual animal response decreased the intensity of the symptoms and the time period needed for their normalization and ensured a safe recovery after the procedure.

Reduction in FS after 6-OHDA administration suggests an abnormality in cardiac contractility. These reductions were mild; even the lowest values were within published normal reference ranges [41], [42], and were not associated at the time with changes in wall thickness. It should be noted that normal ranges of echocardiogram parameters are not well established for macaque monkeys and the limited data are based on single recordings with different types of anesthesia. Because FS measurements are not well established for monkeys, we evaluated the percent change of FS over time for each individual animal. Three animals in this study had recurring declines in the change of FS following 6-OHDA, suggesting that the loss of noradrenergic cardiac innervation may produce reductions in left ventricular systolic function. The animal with the greatest decrease in FS (R04094) uniquely had increased troponin I levels 1 week after toxin suggesting injury to the myocardium. This was coupled with lack of recovery in the LV MHED uptake from 1 week to 4 weeks, perhaps demonstrating a more severe hypertensive response to 6-OHDA. The other animals did not exhibit elevated cardiac troponin I levels and had no or less of an initial decrease in FS accompanied by greater increases in total MHED uptake.

Noninvasive PET imaging of cardiac sympathetic innervation allowed us to monitor over time the cardiac tissue response to 6-OHDA challenge. We used equilibrium DV relative to whole blood to analyze uptake of MHED, because the uptake index conventionally used [20] declined rapidly as progressively later time windows were used for estimation. This behavior, and the success of an analysis method that assumes reversibility of the radioligand uptake, are at least partly due to progressive metabolism of MHED into compounds with no specific affinity to noradrenergic terminals. If blood metabolite analysis is not in the protocol, the DV estimates represent the most reproducible and least arbitrary way to use all the measured data to estimate a single number representing the uptake process. The use of DV – 1 to represent the innervation-specific part of the uptake is based on the observation that lesioning reduced the DV values to ∼1 in all regions in all animals.

Systemic administration of 6-OHDA induced loss of catecholaminergic innervation of the heart (greater loss in inferior LV myocardium), which persisted 3 months after toxin challenge. Although nerve terminals in all regions of the LV reacted similarly to 6-OHDA at 1 week, regional rates of recovery varied over time, with an average 51% remaining deficit after 3 months. The anterior region consistently recovered most rapidly and the inferior wall most slowly. Our findings resemble those in PD: MHED PET established loss in sympathetic myocardial innervation [19] and 6-[^18^F]fluorodopamine imaging suggested preservation in septal [18] and anterior [6] walls of the LV. The nonuniform pattern of MHED uptake may be caused by regional differences in blood perfusion that affected the distribution or metabolism of the neurotoxin. Another possibility is that the subpopulation of ganglionic cells innervating the area has a greater sensitivity to 6-OHDA. It could be argued that the regional recovery was due to re-growth of cardiac muscle tissue, instead of reinnervation. Yet, results from the echocardiogram showed no significant differences in posterior or anterior wall thickness in either diastole or systole. Bai and colleagues [43] have reported recovery of cardiac sympathetic nerves following subcutaneous delivery of 6-OHDA insult in rats. In addition, sympathetic reinnervation has been described after heart transplantation, suggesting that the system has a certain plasticity that could be exploited for regenerative or neuroprotective treatments [44], [45], [46]. Increasing animal numbers would help further define regional differences in LV MHED uptake. Further investigation of the mechanisms of regional loss is needed, as it may facilitate the identification of possible therapeutic targets.

The timeline of our experimental design was based on our previous experience with administration of neurotoxins in the central nervous system of nonhuman primates. For example, an observation period of 3 months after MPTP challenge allows for the dopaminergic nigral cell neurodegeneration to be completed and defines a stable syndrome [33], [47], [48]. Lack of significant changes in MHED uptake between 10 and 14 weeks suggests that as predicted, by 3 months the catecholaminergic lesion was stabilized and recovery mechanisms were completed. The characterization of the timeline for recovery and stabilization of the lesion in this model will be helpful when designing a study to test disease-modifying strategies for the heart. Follow-up experiments with endpoints exceeding 14 weeks after toxin administration would further confirm that 6-OHDA produces a stable lesion in the LV.

Dopamine, epinephrine, norepinephrine, and DOPAC circulating levels were significantly decreased after 6-OHDA, which indicates that the neurotoxin affected peripheral catecholaminergic sources such as adrenal medulla, facilitating onset of cardiac dysautonomia. The positive correlation found between catecholamine levels and cardiac MHED uptake further suggests a similar toxic effect of 6-OHDA in different peripheral catecholaminergic cells. The drop in circulating catecholamines did not seem to affect animal health, probably because of adaptive sympathetic presynaptic supersensitivity [49]. Studies in dogs did not find changes in catecholamine levels, suggesting a species difference in the sensitivity to neurotoxin effects [32].

Cardiac dysautonomia in PD patients is clinically characterized by orthostatic hypotension, an increase in corrected QT intervals (QTc), and reductions in heart rate variability [6], [7], [8], [9], [10], [52], [53]. The presence of these symptoms was not confirmed in this study, because they are detected in an awake state and the cardiac and blood pressure evaluations were performed under anesthesia. Nonuniform cardiac innervation, affects cardiac repolarization and has been associated with arrhythmias [6], [15]. Future preclinical experiments using telemetric measurements of heart rate, blood pressure, and locomotive activity [50], [51] will allow their identification in awake animals. Injections of vasoactive pharmaceuticals such as phenylephrine or sodium nitroprusside may also help characterize the cardiovascular response.

The monkeys presented abnormal feces and weight loss that became significant 4 weeks after 6-OHDA. The frequency of loose stools and diarrhea correlated with the amount of weight loss. Supplementation of feedings with chow soaked in protein-enriched drink (standard practice in our facility for animals loosing weight) enticed feeding, increased fluid intake and helped prevent further weight loss. The abnormal feces could have been the result from stress (each animal underwent procedures at least monthly), but their presence were also described in 6-OHDA-treated dogs that did not undergo those evaluations, suggesting an effect of 6-OHDA in the gastrointestinal tract [31]. The enteric nervous system consists of dopaminergic neurons (myenteric and submucosal plexus), which are potentially susceptible to the toxic effects of systemic 6-OHDA [54]. In that regard, rats treated with 6-OHDA have decreased TH mRNA levels in the duodenum [55]. Reduced sympathetic innervation of the intestinal tract can lead to bowel dysmotility and, therefore, abnormal feces.

Collectively, the changes in cardiac innervation, catecholamine levels and feces suggest that systemic dosing of 6-OHDA affects multiple vulnerable catecholaminergic peripheral cells, and this property can be applied to model dysautonomias in nonhuman primates. Similar to CNS neurotoxic models, a 6-OHDA-induced dysautonomia model presents limitations like acute onset and the risk for spontaneous recovery [39]. Intravenous administration of 6-OHDA does not induce a PD motor syndrome, therefore a comprehensive PD model would require supplementation with systemic MPTP or direct intracerebral dosing of 6-OHDA to induce dopaminergic nigral cell loss. Postmortem analysis and quantification of catecholaminergic cell and terminal loss in susceptible tissues, as well as evaluation of pathologies typical of PD–related neurodegeneration, such as inflammatory cell response and intracytoplasmic accumulation of alpha synuclein [56], are warranted to further characterize the effect of 6-OHDA in peripheral catecholaminergic cells.

To conclude, systemic administration of 6-OHDA to rhesus monkeys mimics features of cardiac dysautonomia in PD that can be tracked and mapped *in vivo* using PET imaging. We hope that these results will facilitate model development to study this symptom and to identify new therapeutic alternatives.
